# Cognition and Other Non-Motor Symptoms in an At-Risk Cohort for Parkinson’s Disease Defined by REM-Sleep Behavior Disorder and Hyposmia

**DOI:** 10.3233/JPD-230285

**Published:** 2024-04-23

**Authors:** Laure Pauly, Armin Rauschenberger, Claire Pauly, Valerie E. Schröder, Gilles Van Cutsem, Anja K. Leist, Rejko Krüger, on behalf of the NCER-PD Consortium

**Affiliations:** a Transversal Translational Medicine, Luxembourg Institute of Health (LIH), Strassen, Luxembourg; b Faculty of Science, Technology and Medicine, University of Luxembourg, Esch-sur-Alzette, Luxembourg; c Translational Neuroscience, Luxembourg Centre for Systems Biomedicine (LCSB), University of Luxembourg, Esch-sur-Alzette, Luxembourg; d Parkinson Research Clinic, Luxembourg, Centre Hospitalier de Luxembourg (CHL), Luxembourg, Luxembourg; e Competence Centre for Methodology and Statistics, Luxembourg Institute of Health (LIH), Strassen, Luxembourg; f Biomedical Data Science, Luxembourg Centre for Systems Biomedicine (LCSB), University of Luxembourg, Esch-sur-Alzette, Luxembourg; g Department of Social Sciences, Institute for Research on Socio-Economic Inequality, University of Luxembourg, Esch-sur-Alzette, Luxembourg

**Keywords:** Parkinson’s disease, cognition, population at risk, quality of life

## Abstract

**Background:**

REM-sleep behavior disorder (RBD) and other non-motor symptoms such as hyposmia were proposed by the Movement Disorder Society as research criteria for prodromal Parkinson’s disease (P-PD). Global cognitive deficit was later added.

**Objective:**

To compare non-motor symptoms, focusing on cognition, between a P-PD group and a matched control group.

**Methods:**

In this cross-sectional, case-control study, in a first set of analyses, we performed extensive cognitive testing on people with (*n* = 76) and a control group without (*n* = 195) probable RBD and hyposmia. Furthermore, we assessed motor and non-motor symptoms related to Parkinson’s Disease (PD). After propensity score matching, we compared 62 P-PD with 62 age- and sex-matched controls. In addition, we performed regression analyses on the total sample (*n* = 271). In a second set of analyses, we used, a.o., the CUPRO to evaluate retrograde procedural memory and visuo-constructive functions.

**Results:**

People with P-PD showed significantly poorer performances in global cognition, visuo-constructive and executive functions, mainly in mental flexibility (*p* < 0.001; *p* = 0.004; *p* = 0.003), despite similar educational levels (*p* = 0.415). We observed significantly more motor and non-motor symptoms (*p* < 0.001; *p* = 0.004), higher scores for depression (*p* = 0.004) and apathy (*p* < 0.001) as well as lower quality of life (*p* < 0.001) in P-PD.

**CONCLUSIONS:**

Our findings confirm that global cognitive, executive, and visuo-constructive deficits define the P-PD group. In addition, depression, apathy, and lower quality of life were more prevalent in P-PD. If replicated in other samples, executive and visuo-constructive deficits should be considered in non-motor P-PD. Determining specific patterns will support early recognition of PD, secondary prevention of complications and the development of neuroprotective treatments.

## INTRODUCTION

Parkinson’s disease (PD) is a neurodegenerative disorder with increasing prevalence. It is mostly diagnosed when more than 60% of the dopaminergic neurons are degenerated and first motor manifestations, such as tremor, rigidity, and slowness of movement, appear [1, 2]. The period between the onset of neuronal degeneration, where symptoms and signs are present, but yet insufficient to define the disease, and the clinical diagnosis is called the prodromal or pre-motor phase and can start up to 20 years before the onset of motor parkinsonism [3, 4]. Given that diagnosing PD means identifying an already advanced neurodegeneration, it is essential to focus on its early detection, e.g. by defining patterns of cognitive and other non-motor symptoms.

Research criteria for prodromal PD (P-PD) were proposed by the Movement Disorder Society (MDS) [5, 6]. Their findings suggest that polysomnographically proven REM-sleep behavior disorder (RBD), abnormal dopaminergic brain imaging (PET/SPECT), subthreshold motor parkinsonism and olfactory dysfunction are the prodromal markers with the highest likelihood to predict α-synucleinopathies, such as PD. Global cognitive deficit was only later added as a criterion for prodromal PD [6].

Cognitive impairment already defines the early stages of PD. In the Luxembourgish PD cohort, approximately 45% of newly diagnosed typical PD patients (disease duration ≤1 year) presented cognitive impairment (Montreal Cognitive Assessment (MoCA)<26). These findings are in line with previous observations of 24 to 54% of cognitive impairment in newly diagnosed PD [6–9]. These deficits may precede clinical PD diagnosis by up to 5 years [3]. Longitudinal studies comparing converters to non-converters describe a prevalence of 42% of cognitive impairment at baseline [7]. Knowledge on the nature of these prodromal cognitive changes is still limited, probably due to the novelty of the concept. Recent studies on cognitive deficits in prodromal PD described that global cognition and diverse cognitive sub-domains, mainly executive functions, less frequently visuospatial functions, memory and language, may be prodromal cognitive features of PD [8–11]. The available studies are very heterogeneous in their study designs (e.g., recruitment strategies), study populations (e.g., age, education), neuropsychological assessments and the tested cognitive domains, complicating the comparability of results [12–14]. Therefore, following previously published recommendations [5, 6, 12, 13], results on cognition in prodromal PD need validation 1) in a deep-phenotyped population, 2) combining predefined prodromal markers, 3) on normative-controlled cognitive data, 4) based on a broad variety of commonly used cognitive assessment tools to evaluate both global cognition and domain-specific cognition, and 5) with at least two tests per cognitive domain.

In the present cross-sectional, case-control study, we performed extensive cognitive testing in an at-risk group for developing PD, defined by probable RBD and hyposmia and compared them with an age- and sex-matched control group. Besides testing different cognitive functions, we investigated additional features such as non-motor (e.g., psychological factors and quality of life) and motor symptoms.

The main aim of this study was to describe non-motor symptoms in P-PD and to define its specific profile focusing on cognition. In the future, participants will be followed-up yearly to capture possible phenoconversion from P-PD to PD, allowing us to determine specific patterns supporting the definition of further possible prodromal markers. Early recognition of PD could not only allow better prognosis but also help the development of neuroprotective therapies.

## METHODS

### Participants

All participants were recruited from the Luxembourg Parkinson Study of the National Centre of Excellence in Research on Parkinson’s disease (NCER-PD). NCER-PD is a monocentric, observational, longitudinal prospective study including a PD, an enriched P-PD as well as a control cohort from Luxembourg and the Greater Region [15], an area of cross-border cooperation between Luxembourg, Germany, Belgium, and France. All participants provided informed consent according to the Declaration of Helsinki. The study is approved by the National Ethics Board (CNER Ref: 202001/03 and 201407/13). The detailed study design, recruitment and screening steps have been described elsewhere [15, 16].

The classification of probable RBD (pRBD) was based on the RBD Screening Questionnaire (RBDSQ score ≥ 7 [17]). The Brief Smell Identification Test (A) (B-SIT) [18] or Sniffin’Stick Identification Test [19] (B-SIT score < 8 [18] or Sniffin’Stick score≤12 [20]) were used to assess olfaction. Each subject underwent a detailed neurological examination by a physician trained in movement disorders and provided information on probable symptoms and disease history. The Movement Disorder Society - Unified Parkinson’s Disease Rating Scale (MDS-UPDRS) [21] was used to assess motor and non-motor symptoms. Inclusion criteria were age 18 years or older and ability to sign the written informed consent. People with PD or other known neurological diseases as well as participants with a history of severe psychiatric disorders were excluded (Supplementary Figure 1).

### Approach

We defined two sets of analyses: The first set of analyses (Set 1) (Flowchart, Supplementary Figure 1A, B), capitalized on the extensive neuropsychological assessment. We adjusted for the effects of other variables (and tested the effect of the variable of interest) with 1) propensity score matching (followed by testing whether the outcome differs between the two groups) and 2) multiple regression (followed by testing the effect of the group on the outcome).

For the second set of analyses (Set 2) (Flowchart, SupplementaryFigure 1C), we compared cognitive performances measured by the CUPRO evaluation system [22]. The size of the P-PD and matched control sample differ slightly between sets, since CUPRO was more recently added to the neuropsychological assessment.

### Neuropsychological assessments

For the first set of analyses, study participants underwent detailed neuropsychological assessments, selected previously based on recommendation by Goldman and colleagues [23] (Table 1). Cognitive measures were combined to evaluate global cognition and the following five cognitive domains: memory, processing speed, executive functions, language, and visuospatial functions.

**Table 1:** Neuropsychological assessments and measured cognitive functions

Cognitive functions	Assessments
Global cognition	Montreal Cognitive Assessment (MoCA)	[24]
Memory
Auditory short-term memory	Digit Span –Forward	[43]
Auditory working memory	Digit Span –Backward	[43]
Visuo-spatial short-term memory	Corsi Block Tapping Task –Forward	[44]
Visuo-spatial working memory	Corsi Block Tapping Task –Backward	[44]
Episodic verbal long-term memory	CERAD Word List Delayed Recall	[45]
Learning ability	CERAD Word List Learning	[45]
Processing speed
Psychomotor speed, Initiation	Trail Making Test (TMT) –Part A	[25]
Processing speed	Stroop Test –Word Reading	[46]
Executive functions
Mental flexibility, Shifting	Trail Making Test (TMT) –Part B &Delta-TMT^*^	[25]
Inhibitory control	Stroop Test –Interference Score	[25, 46]
Dysexecutive syndrome	Frontal Assessment Battery (FAB)	[47]
Mental flexibility	Isaacs Set Test	[48]
Language
Language –Denomination	Boston Naming Test –short form	[45]
Fluency, Word initiation	Semantic Fluency (animals, 2 min)	[25]
	Phonemic Fluency (letter “F”, 1 min)	[24]
Visuospatial functions
Visuoconstructive capacities	Qualitative Scoring MMSE Pentagon	[49]
	Cube Copying Task	[24]
Visuospatial judgment	Benton Judgment of Line Orientation (JLO)	[50]

N.B.: We assigned cognitive tests to specific cognitive domains. Given that no cognitive assessment evaluates purely one cognitive function, overlap cannot be excluded. ^*^Delta-TMT is defined as (TMT-B) –(TMT-A).

For the second set of analyses, we applied the CUPRO, (short for CUbe drawing PROcedure) evaluation system to assess the cube copying procedure (Intermediate Score 1 –CUPRO-IS1), representing retrograde procedural memory and the final result of the cube (Intermediate Score 2- CUPRO-IS2), representing visuo-constructive functions [22]. Furthermore, MoCA [24] and Trail-Making-Test [25] were also assessed in this set of analyses.

Mild and severe cognitive impairment were defined as impaired global cognition based on MoCA <26 and < 21, respectively [24, 26]. Participants with MoCA ≥26 were classified as cognitively normal.

### Self-assessment questionnaires

The Beck Depression Inventory-I (BDI-I) [27], the Starkstein Apathy Scale (SAS) [28], and the Parkinson’s Disease Questionnaire (PDQ-39) [29] were applied to assess symptoms of depression and apathy, and quality of life, respectively. Participants reported non-motor and motor aspects of experiences of daily living in the MDS-UPDRS Part I and II.

### Statistics

Two different statistical methods were used to adjust for the effects of potential confounders, namely propensity score matching and multiple regression.

• In a first step we chose to test differences between samples, both groups were matched by age and sex (propensity score matching; matching tolerance = 0.05). As many outcomes are not normally distributed, differences in demographic and clinical characteristics as well as cognitive performance between the groups were analyzed using the Mann-Whitney U test (two-tailed) for numerical variables (which might be non-normally distributed) and Pearson’s chi-squared test (two-tailed) for binary variables (Supplementary Figure 1A, C; Tables 2 and 3; Supplementary Tables 1 and 2). We corrected for multiple testing using the Bonferroni correction (*p*≤0.05/n, *n* = number of comparisons) (**).
• To validate the findings in a larger sample and to further assess the relationship between the groups and demographic, clinical and cognitive factors, controlled for sex, age and education depending on P-PD status, we applied, in a second step, multiple linear and logistic regressions (Supplementary Figure 1B; Tables 4 and 5). The significance threshold was set up at p-value≤0.05. We corrected for multiple testing (*p*≤0.05/n, *n* = number of comparisons) (**).

**Table 2:** Demographical and clinical information for prodromal PD (P-PD) and control group

Variable	Descriptive statistics						*p*
	Prodromal PD			Control			Prodromal PD
	*n* = 62			*n* = 62			vs. Control
	*Mean*	*SD*	*n*	*Mean*	*SD*	*n*
Sex, M/F	37/25		62	38/24		62	*p* = 1.000
Age, y	63.52	5.96	62	63.70	8.04	62	*p* = 0.793
RBDSQ (/13)	9.07	1.83	62	2.00	1.79	62	*p* < 0.001
Sniffin’Stick (/16)	10.11	3.19	53	14.18	1.06	62	*p* < 0.001
BSIT-A (/12)	6.48	1.94	61	NA	NA	0	NA
Education, y	13.00	4.25	60	13.76	3.66	62	*p* = 0.415
MDS-UPDRS I (/32)	8.22	5.96	46	4.65	4.49	62	*p* < 0.001^**^
MDS-UPDRS II (/32)	2.68	4.01	57	1.10	1.77	62	*p* = 0.004^**^
MDS-UPDRS III (/132)	4.71	6.57	59	4.19	5.06	59	*p* = 0.872
BDI-I (/63)	8.69	6.89	54	5.34	5.13	62	*p* = 0.004^**^
SAS (/42)	13.56	5.32	55	9.93	5.09	61	*p* < 0.001^**^
PDQ-39 (%)	13.32	12.63	53	5.87	6.15	61	*p* < 0.001^**^

Both groups were defined on RBDSQ, Sniffin’Stick and BSIT-A and matched for sex and age. SD, standard deviation; M, male; F, female; n, sample size; MDS-UPDRS, Movement Disorder Society –Unified Parkinson’s Disease Rating Scale; RBDSQ, REM Sleep Behavior Disorder (RBD) Screening Questionnaire; BSIT, Brief Smell Identification Test; BDI-I, Beck Depression Inventory; SAS, Starkstein Apathy Scale; PDQ-39, Parkinson’s disease questionnaire 39-item. ^*^Significant at the unadjusted 5% level (*p*≤0.05) (two-tailed); ^**^Significant at the Bonferroni-adjusted 5% level (*p*≤0.05/7) (two-tailed).

**Table 3:** Results of neuropsychological assessments for prodromal PD (P-PD) compared to the control group

Variable	Descriptive statistics						Significance
	Prodromal PD			Control			Prodromal PD
	*n* = 62			*n* = 62			vs. Control
	*Mean*	*SD*	*n*	*Mean*	*SD*	*n*
Montreal Cognitive Assessment (MoCA) (/30)	26.05	2.47	62	27.39	2.43	62	*p* < 0.001^**^
Trail-Making-Test Part A (TMT-A) (s)	45.13	37.52	61	44.73	36.94	62	*p* = 1.000
Trail-Making-Test Part B (TMT-B) (s)	111.7	61.77	61	89.66	38.50	62	*p* = 0.030^*^
Delta-TMT (TMT-B) –(TMT-A)	66.52	49.74	61	44.94	23.30	62	*p* = 0.003^**^
Digit Span Test Forwards (/16)	8.61	1.73	62	8.47	1.66	62	*p* = 0.668
Digit Span Test Backwards (/14)	5.90	1.63	62	6.16	1.87	62	*p* = 0.651
Corsi Block-Tapping Test Forward (/16)	8.21	1.50	62	8.00	1.34	61	*p* = 0.450
Corsi Block-Tapping Test Backward (/14)	7.74	1.59	62	7.59	1.94	61	*p* = 0.628
Kaplan Stroop Interference Score (s)	64.90	35.97	59	52.35	22.99	62	*p* = 0.146
Semantic Fluency Test (N Letter F, 1 min)	10.37	4.43	60	11.45	4.50	62	*p* = 0.307
Phonemic Fluency Test (N Animals, 1 min)	29.07	9.62	61	30.41	7.10	61	*p* = 0.258
Isaacs Set Test (N)	32.47	7.58	58	34.98	6.09	58	*p* = 0.058
Interlocking Pentagons Test (incorrect/correct)	2/60		62	4/58		62	*p* = 0.676
Cube Copying Task (incorrect/correct)	24/38		62	9/53		62	*p* = 0.004^*^
Benton’s Judgment of Line Orientation Test (/30)	24.11	4.88	61	24.45	4.35	62	*p* = 0.877
Frontal Assessment Battery (FAB) (/18)	15.48	2.17	52	16.21	1.53	62	*p* = 0.091
CERAD Word list (Learning) (/30)	22.60	3.93	62	23.05	3.29	62	*p* = 0.722
CERAD Word list (Delayed Recall) (/10)	7.10	2.28	60	7.59	1.77	61	*p* = 0.327

SD, standard deviation; CERAD, Consortium to Establish Registry for Alzheimer Disease. ^*^Significant at the unadjusted 5% level (*p*≤0.05) (two-tailed); ^**^Significant at the Bonferroni-adjusted 5% level (*p*≤0.05/18) (two-tailed).

To evaluate the assumptions of the linear and logistic regressions, we confirmed in a first step that the samples are independent; No participant was included twice or more and they have not been measured under two or more conditions. However, we cannot exclude that we might have included participants that share a family link. In a second step, we assessed the variance inflation factor (VIF), measuring of how much the variance of the estimated regression coefficients increases due to multicollinearity. We could not detect any VIF greater than 2 and excluded therefore multicollinearity. To verify the linearity assumption, we examined scatter plots of the residuals against the predictors. As we did not observe any relationship between the residuals and the predictors, we have no evidence of any non-linear effects. To verify the linearity assumption for the logistic regressions we plotted the partial residuals against predictors and observed a linear relationship between each predictor variable and the log-odds of the response variable.

All statistical analyses were performed using R version 4.2.0 GUI 1.78 and RStudio version 2023.03.1 + 446.

## RESULTS

For Set 1, in total, 271 participants fulfilled the inclusion criteria, 76 participants with probable RBD and hyposmia and 195 control subjects without RBD and without hyposmia (Supplementary Figure 1A, B).

### Propensity score matching

After matching for age and sex, we compared 62 P-PD participants with 62 control subjects (Supplementary Figure 1A; Tables 2 and 3). Confirming successful matching, the groups did not differ significantly in sex (*p* = 1.000) or age (*p* = 0.793). Furthermore, they did not differ on years of education (*p* = 0.415). After multiple testing correction, the P-PD group presented significantly higher scores in SAS (*p* < 0.001), BDI-I (*p* = 0.004), MDS-UPDRS I & II (*p* < 0.001, *p* = 0.004, respectively), and a significantly lower score for PDQ-39 (*p* < 0.001) compared to the matched control subjects.

Significant group differences were found in cognition (Table 3). The P-PD group presented significantly lower scores in MoCA (*p* < 0.001) and Delta-TMT scores (*p* = 0.003) compared to the control group. We observe a tendency for deficits in the Cube Copying Task in the P-PD, however the difference is not significant after correction for multiple testing.

When investigating the distribution of the total MoCA score, we observed that 53/62 (85%) and only 37/62 (60%) participants presented normal cognition (based on MoCA ≥ 26 [24]) in the control group, respectively the P-PD group; 7/62 (11%) and 23/62 (37%) participants presented mild cognitive impairment (MCI; based on 21 > and MoCA < 26) in the control group [24], respectively the P-PD group. Furthermore, 2/62 (3%) and 2/62 (3%) participants presented severe cognitive impairment (based on MoCA < 21 [26]) in the control group, respectively the P-PD group.

### 3.2 Regressions

After adjusting for age, sex, and education as well as multiple testing correction, the P-PD group was associated with significantly different scores on MoCA, TMT-B, Delta-TMT, Cube Copying Task, BDI, SAS, PDQ-39 as well as on the MDS-UPDRS I and II. Furthermore, nominal significant different scores were observed for the Stroop Interference Score, FAB and Isaacs Set test (Tables 4 and 5).

**Table 4:** Regression analyzing the relationship between the groups and the demographical and clinical factors, controlled for age, sex and education

Dependent variables		Independent variables			
		Age	Sex	Education	Prodromal PD
MDS-UPDRS I	Estimate	0.042	1.457	–0.019	3.491
	*p*	*p* = 0.159	*p* = 0.018^*^	*p* = 0.811	*p* < 0.001^**^
MDS-UPDRS II	Estimate	0.021	–0.087	–0.082	1.462
	*p*	*p* = 0.199	*p* = 0.781	*p* = 0.043^*^	*p* < 0.001^**^
MDS-UPDRS III	Estimate	0.118	–0.736	–0.138	0.807
	*p*	*p* < 0.001^**^	*p* = 0.222	*p* = 0.074	*p* = 0.227
BDI	Estimate	0.018	2.050	–0.066	2.931
	*p*	*p* = 0.601	*p* = 0.004^*^	*p* = 0.462	*p* < 0.001^**^
SAS	Estimate	0.032	–0.017	–0.316	2.957
	*p*	*p* = 0.307	*p* = 0.979	*p* < 0.001^**^	*p* < 0.001^**^
PDQ-39	Estimate	–0.020	1.085	–0.260	6.043
	*p*	*p* = 0.718	*p* = 0.318	*p* = 0.059	*p* < 0.001^**^

^*^Significant at the unadjusted 5% level (*p*≤0.05) (two-tailed); ^**^Significant at the Bonferroni-adjusted 5% level (*p*≤0.05/23) (two-tailed).

**Table 5:** Regression analyzing the relationship between the groups and the cognitive factors, controlled for age, sex, and education

Dependent variables		Independent variables			
		Age	Sex	Education	Prodromal PD
MoCA	Estimate	–0.033	0.463	0.191	–1.166
	*p*	*p* = 0.015^*^	*p* = 0.089	*p* < 0.001^**^	*p* < 0.001^**^
TMT-A	Estimate	0.642	6.490	–0.480	1.401
	*p*	*p* = 0.002^**^	*p* = 0.107	*p* = 0.351	*p* = 0.751
TMT-B	Estimate	1.016	–3.057	–3.323	23.380
	*p*	*p* < 0.001^**^	*p* = 0.541	*p* < 0.001^**^	*p* < 0.001^**^
(TMT-B) –(TMT-A)	Estimate	0.374	–9.547	–2.843	21.978
	*p*	*p* = 0.091	*p* = 0.032^*^	*p* < 0.001^**^	*p* < 0.001^**^
Digit Span Test Forward	Estimate	–0.019	–0.382	0.006	–0.112
	*p*	*p* = 0.096	*p* = 0.093	*p* = 0.836	*p* = 0.655
Digit Span Test Backwards	Estimate	–0.025	–0.070	0.066	–0.113
	*p*	*p* = 0.027^*^	*p* = 0.754	*p* = 0.020^*^	*p* = 0.646
Corsi Block Tapping Forward	Estimate	–0.033	–0.047	0.040	0.311
	*p*	*p* < 0.001^**^	*p* = 0.799	*p* = 0.086	*p* = 0.124
Corsi Block Tapping Backward	Estimate	–0.049	–0.445	0.115	0.464
	*p*	*p* < 0.001^**^	*p* = 0.057	*p* < 0.001^**^	*p* = 0.071
Stroop Interference Score	Estimate	0.842	3.289	–0.577	9.170
	*p*	*p* < 0.001^***^	*p* = 0.307	*p* = 0.162	*p* = 0.011^*^
Semantic Fluency	Estimate	–0.152	1.171	0.431	–0.851
	*p*	*p* = 0.005^*^	*p* = 0.273	*p* = 0.002^**^	*p* = 0.470
Phonemic Fluency	Estimate	–0.037	0.809	0.235	–0.369
	*p*	*p* = 0.165	*p* = 0.131	*p* < 0.001^**^	*p* = 0.533
Isaacs Set Test	Estimate	–0.139	1.313	0.404	–2.354
	*p*	*p* < 0.001^**^	*p* = 0.107	*p* < 0.001^**^	*p* = 0.009^*^
Interlocking Pentagons	Estimate	–0.050	–0.872	0.212	0.951
	Odds Ratio	0.952	0.418	1.236	2.587
	*p*	*p* = 0.190	*p* = 0.226	*p* = 0.019^*^	*p* = 0.272
Cube Copying Task	Estimate	–0.008	–0.403	0.198	–1.235
	Odds Ratio	0.992	0.668	1.219	0.291
	*p*	*p* = 0.706	*p* = 0.255	*p* < 0.001^**^	*p* < 0.001^**^
Benton JLOT	Estimate	–0.075	–3.619	0.310	–0.688
	*p*	*p* = 0.003^*^	*p* < 0.001^**^	*p* < 0.001^**^	*p* = 0.212
FAB	Estimate	–0.032	0.212	0.130	–0.688
	*p*	*p* = 0.002^**^	*p* = 0.318	*p* < 0.001^**^	*p* = 0.005^*^
CERAD Word List learning	Estimate	–0.088	2.108	0.172	–0.021
	*p*	*p* < 0.001^**^	*p* < 0.001^**^	*p* = 0.002^**^	*p* = 0.965
CERAD Word List Delayed Recall	Estimate	–0.040	0.982	0.095	–0.083
	*p*	*p* < 0.001^**^	*p* < 0.001^**^	*p* = 0.001^**^	*p* = 0.739

Regression analyzing the relationship between the groups (N prodromal PD = 76; N control group = 195) and the cognitive factors, controlled for age, sex, and education. Multiple logistic regression for “Interlocking Pentagons” and “Cube Copying Task” otherwise multiple linear regression. ^*^Significant at the unadjusted 5% level (*p*≤0.05) (two-tailed); ^**^Significant at the Bonferroni-adjusted 5% level (*p*≤0.05/23) (two-tailed).

Both analytical strategies (Supplementary Figure 1A, B) yielded consistent results: Both sets of findings indicate impaired global cognition, executive and visuo-constructive functions in the P-PD group compared to the matched control group. With the matching analyses (Supplementary Figure 1A), significantly lower performances in executive functions were only observed in one cognitive test (Trail-Making-Test, TMT); the difference in visuo-constructive abilities was only nominally significant. However, in the regression analyses (Supplementary Figure 1B), taking the total sample into consideration, we observed that several cognitive assessments measuring executive functions were nominally significant impaired in the P-PD group compared to the matched control group (Stroop Interference Score; Isaacs Set Test; Frontal Assessment Battery, FAB), which were however not significant after Bonferroni correction (*p* = 0.011, *p* = 0.009, *p* = 0.005, respectively). Differences in visuo-constructive abilities were significant in the larger sample (*p* < 0.001). Furthermore, in both analyses, scores for depression (BDI-I), apathy (SAS), motor and non-motor symptoms (MDS-UPDRS I and II) were significantly higher and the score for quality of life (PDQ-39) was significantly lower in P-PD.

Results of the Set 2 are presented in the Supplementary Tables 1 and 2.

## DISCUSSION

The aim of the present study was to investigate non-motor symptoms focusing on the cognitive profile in a prodromal PD (P-PD) cohort with self-assessments and extensive cognitive testing. We compared presence and level of non-motor symptoms, focusing on cognition, between P-PD and age- and sex-matched control subjects. The present study demonstrates that cognitive performance was impaired in the at-risk group for developing PD compared to the control group. More precisely, participants with P-PD present significantly lower scores in global cognition, executive and visuo-constructive functions (in tasks with higher complexity) compared to the matched control group. In addition, we observed significantly more difficulties in motor and other non-motor symptoms of experiences of daily living, as well as significantly higher scores for depression and apathy and significantly lower scores for quality of life in the P-PD group.

The observation of global cognitive and executive deficits are consistent with the conclusion of recently published studies stating that these cognitive impairments may be prodromal features of PD [8–10, 30]. With the screening tool for global cognition (MoCA) employed here, we observed that 40% of the at-risk group presented cognitive impairment, in contrast to only 15% in the matched control group. These results are consistent with findings of 42% with MCI and global cognitive deficit at baseline in a longitudinal study comparing converters to non-converters [7] and close to the prevalence of 45% of MCI in newly diagnosed PD patients in our PD cohort [15]. A longitudinal study on RBD P-PD participants found that global cognitive deficits appeared approximately 5 years before phenoconversion to PD compared to age- and sex-matched control subjects [3].

We found tendencies for impaired executive functions in P-PD across all assessments previously defined to evaluate these functions. After correction for multiple testing, significant differences remained for one sub-domain of executive functions: mental flexibility, as measured by the Trail-Making-Test (TMT). Therefore, we carefully interpret that, out of a range of neuropsychological assessments of executive functioning, the TMT might be the most sensitive for detecting executive changes in P-PD. Our findings of significantly impaired mental flexibility and a trend towards impairment in other sub-domains of executive functions are in line with observations in longitudinal and cross-sectional studies in prodromal PD cohorts [8, 9, 11, 30, 31]. Furthermore, they match with the cognitive profile of an executive deficit in newly diagnosed PD patients [32] and its association to the frontal lobe and modulation by dopaminergic input [33]. Our observations of early pre-diagnostic impairment of executive functions lend support to the “Dual Syndrome Hypothesis” [34], describing the possibility of two sub-types of cognitive impairment in PD; the “frontal-striatal subtype”, defined by predominant executive deficits related to increased dopaminergic loss starting early in the disease progression; and the “posterior and temporal subtype” with predominant visuospatial, memory and language deficits, related to increased cholinergic loss [34]. In the present study, we did not find significant differences in processing speed, language, learning and memory. Only a few studies tested learning and memory [10–13, 30]. Memory impairments have been observed in patients who converted to PD within 2 years but not in the earlier prodromal stages [35]. Based on the results in Set 2, by applying the CUPRO evaluation system [22], we confirmed that the significant difficulties observed in the Cube Copying Task (initial scoring on 1 point [24]) are due to visuo-constructive deficits and not to a deficit of retrograde procedural memory. To our knowledge, the current study is the first to systematically evaluate retrograde procedural memory in P-PD. Given that, in our previous work on retrograde procedural memory in already diagnosed PD, we did not see any significant correlation between this memory concept and disease duration [22], we assumed that retrograde procedural memory might be already impaired in the prodromal stages of PD.

The combination of the absence of memory and language impairment, the visuo-constructive impairment only in the more complex assessments and the previously discussed point of a more “frontal-striatal subtype” suggest that the sample may still be in the early stage of P-PD. Previous findings demonstrated that motor variables have been found to be highly predictive of the phenoconversion to parkinsonism [36]. The fact that we did not see any significant differences for the measured motor assessments (UPDRS part III) and that the UPDRS score is estimated to become only abnormal at 4.5 years before diagnosis [37] are further arguments highlighting the possibility that the cohort is in the early stage of P-PD. This might be explained by our wide study design defining the prodromal cohort based on a population-wide participant recruitment in which we invited the entire Luxembourgish population between 55 and 75 years to participate in an online survey about their sleep quality and possible sleeping difficulties. One additional fundamental strength of the current study is the thorough recruitment steps and deep phenotyping of the participants, involving a complex study design, including self-reported, web-based questionnaires, telephone interviews, face-to-face assessments, and longitudinal follow-up assessments. Moreover, by combining two validated prodromal markers, RBD and hyposmia, we work on a population that is at high risk to develop PD. Furthermore, as recommended [12], we assured that the groups were well described and matched for possible confounding factors such as age and sex. Domain-specific cognitive deficits were investigated each through several assessments, allowing us to cross-validate our results. While global cognition and executive functions have been frequently evaluated, learning, memory, visuo-spatial cognition, and language abilities are less frequently assessed [12, 13]. Although we administered an extensive range of neuropsychological assessments, we acknowledge that no cognitive assessment evaluates purely one cognitive function and impairments in one domain may be reflected in impaired performances on tests assessing other domains. Lastly, we cannot fully exclude the possibility of cognition in P-PD being affected by sleep problems and depressive mood. Sleep abnormalities and mood disorders such as depression are validated signs for P-PD [5, 6]. We repeated our regressions by additionally controlling for apathy and depression and the adjustment for apathy and depression does not change our conclusions on the effects of the disease status on the outcomes (significant vs. insignificant). Given that the study participants are characterized with sleep abnormalities and knowing that sleep quality plays a crucial role in the well-functioning of cognition, cognitive performance may be affected by these confounders [38].

Our study has the limitation that it focuses on an at-risk cohort based on probable RBD (pRBD) and not on a polysomnographically proven idiopathic RBD (iRBD). According to the MDS criteria for P-PD, iRBD based on polysomnography has a positive likelihood ratio of 130 compared to only 2.8 for the questionnaire-based pRBD [6]. Therefore, to follow the gold standard for RBD diagnosis and to enrich the prodromal cohort, participants with pRBD are currently undergoing video-polysomnography to confirm the diagnosis of RBD. Furthermore, given that our P-PD cohort is defined by pRBD and hyposmia, we need to highlight that our observations focus mainly on one specific subtype of P-PD, as we do not control for all potential prodromal markers and that it cannot be generalized on all the P-PD subtypes. Especially because iRBD in PD has been associated with higher burden of non-motor symptoms, such as impaired cognitive functions [36, 39]. In order to address the heterogeneity in P-PD, we are currently working on the investigation of a population based on additional prodromal signs, e.g.: by combining alternative prodromal signs, such as constipation and genetic predispositions [40]. Exploring different markers may yield valuable insights, particularly if they are associated with distinct cognitive patterns and varying degrees of severity [13]. Another limitation lies in the fact that not all participants with RBD might develop PD, as RBD is also a risk factor for other synucleinopathies, such as dementia with Lewy bodies or multiple system atrophy [41]. The understanding of the heterogeneity in P-PD is essential to understanding the diversity of clinical PD and the mechanisms behind this variability.

The trends described in the present study highlight the importance of investigating cognitive performances and other non-motor symptoms in populations at risk of developing Parkinson’s disease. However, as these findings are based on the cross-sectional analyses it needs validation on longitudinal observations. Therefore, we aim to confirm the reported findings through longitudinal follow-up, currently foreseen. Furthermore, for future projects, it would be interesting to also include subjective cognitive decline in P-PD, as little knowledge on prevalence and progression of subjective cognitive decline exists in P-PD [13], and to compare different risk factor profiles involved in P-PD.

In conclusion, our findings confirm that global cognitive, executive, and visuo-constructive deficits are more prevalent in individuals at risk for PD based on probable RBD and hyposmia. In addition, people with P-PD had significantly more self-reported motor and other non-motor symptoms, such as depression and apathy, and a lower quality of life. The validation of these results, on normative-controlled, extensive cognitive and clinical data in a deep-phenotyped population is essential, as knowledge on the nature of these cognitive changes is still limited due to the novelty of the concept of cognitive deficits in P-PD [14]. Combining non-motor prodromal signs, including global cognition, executive and visuo-constructive functions, depression, apathy, and quality of life may improve the description of the P-PD phenotype and allow a clearer identification of the at-risk population for PD. Based on our findings and if replicated in other samples, we suggest considering the addition of executive and visuo-constructive deficits as a non-motor sign in P-PD. A clear definition of the P-PD phenotype will have an important impact when disease-modifying treatments will become available [42].

## Supplementary Material

Supplementary Material

## Data Availability

The dataset used for this manuscript is available upon request to the Data and Sample Access Committee of the Luxembourg Parkinson’s Study (via email: request.ncer-pd@uni.lu).
