# Supplementary material for: Cognition and Other Non-Motor Symptoms in an At-Risk Cohort for Parkinson’s Disease Defined by REM-Sleep Behavior Disorder and Hyposmia
Source: J Parkinsons Dis. 2024 Apr 23;14(3):545–56. doi: 10.3233/JPD-230285 (PMC11091555; doi:10.3233/JPD-230285)
Supplement: Supplementary Material [file jpd-14-jpd230285-s001.pdf]

Supplementary Material

Cognition and Other Non-Motor Symptoms in an At-Risk Cohort for Parkinson's Disease Defined by REM-Sleep Behavior Disorder and Hyposmia

Supplementary Figure 1. Figure representing inclusion/exclusion criteria, the propensity score (PS) matching (A) and regression (B) steps for both groups, prodromal PD, and control group.

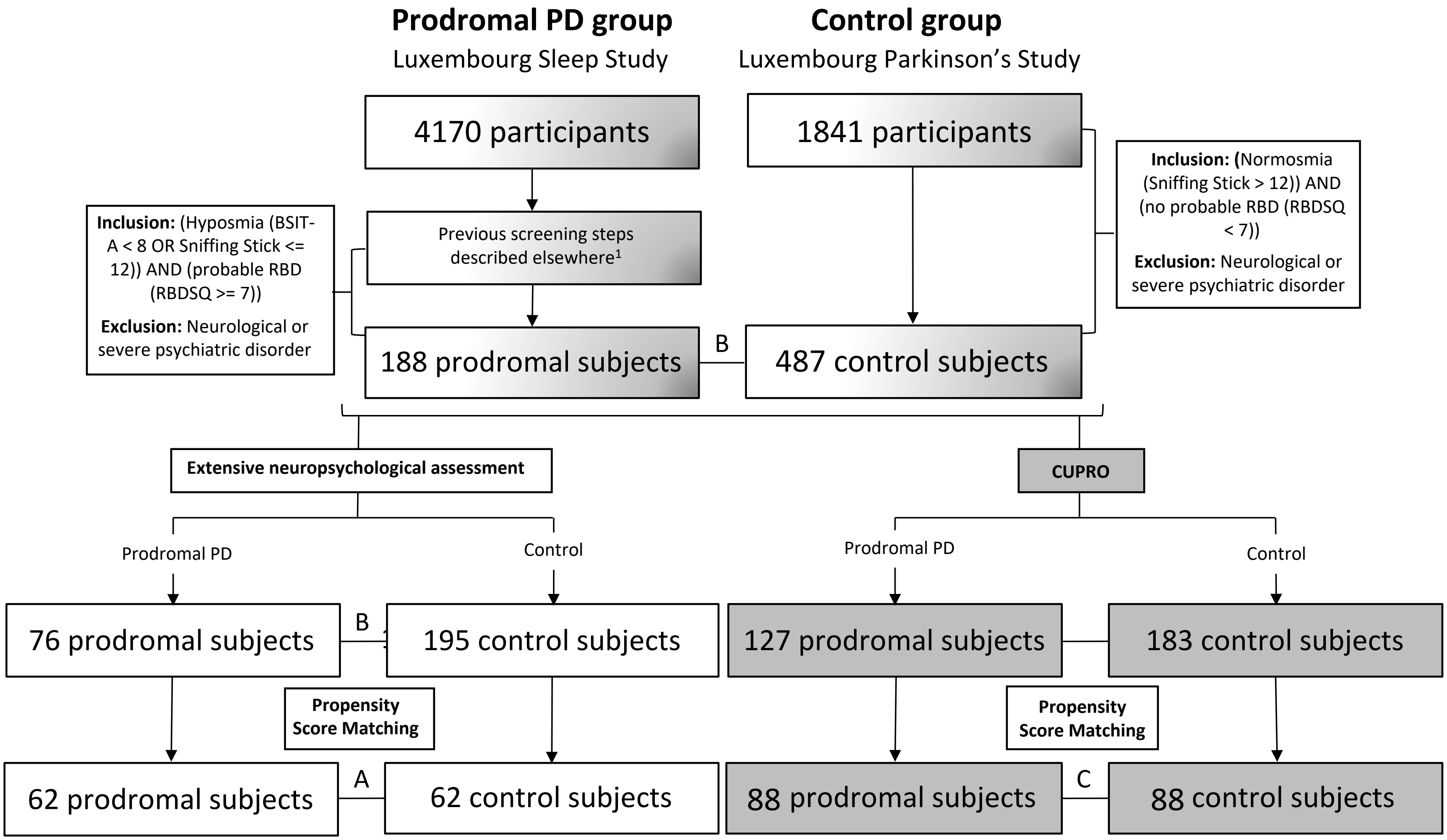

The main analyses on the extensive cognitive assessment are represented in green, the supra-analyses on a reduced cognitive assessment including the novel assessment the CUPRO is represented in orange. [1]McIntyre et al. 2023 (in preparation)

Set 2

In the second set of the analyses (see Flowchart, Supplementary Figure 1), we compared cognitive performances measured by our novel assessment, the CUPRO evaluation system [1]. With the CUPRO evaluation system, we assessed our main outcome variables for this set, the Cube copying procedure (Intermediate Score 1 - IS1), representing retrograde procedural memory and the final result of the Cube (IS2), representing visuo-constructive functions [1]. Given that the CUPRO assessment tool has only been recently developed and integrated in the Luxembourg Parkinson's Study, not all the control participants that have participated in the extensive cognitive testing session (supplementary visit at the research clinic) have a CUPRO evaluation from their cube drawing. Therefore, we decided to observe this variable in an additional analyses, so that by filtering for this assessment, we do not impact the power of our main analyses on the broad cognitive assessment in P-PD.

Supplementary Table 1. Demographical and clinical information for prodromal PD (P-PD) and control group. Both groups were defined on RBDSQ, Sniffin'Stick, and BSIT-A and matched for sex and age.

Variable	Descriptive statistics						p	
	Prodromal PD n = 88			Control n = 88			Prodromal PD vs. Control	
	Mean	*SD*	*n*	*Mean*	*SD*	*n*		
**Sex, M / F**	47/41		88	47/41		88	p = 1.000	
**Age, y**	64.98	5.76	88	64.59	5.86	88	p = 0.648	
**RBDSQ (/13)**	9.03	1.62	88	2.47	1.72	88	p < 0.001	
**Sniffin'Stick (/16)**	9.91	3.18	78	13.94	1.03	88	p < 0.001	
**BSIT-A (/12)**	6.64	2.04	88	NA	NA	0	NA	
**Education, y**	13.11	4.72	80	14.53	4.07	86	p = 0.058	
**MDS-UPDRS I (/32)**	8.37	6.21	35	4.81	3.89	87	p = 0.002	**
**MDS-UPDRS II (/32)**	3.27	4.92	45	0.82	1.28	88	p < 0.001	**
**MDS-UPDRS III (/132)**	5.96	8.55	49	4.35	4.10	82	p = 0.833	
**BDI-I (/63)**	9.24	8.08	46	5.18	4.74	88	p = 0.001	**
**SAS (/42)**	13.29	5.59	45	9.51	4.26	88	p < 0.001	**
**PDQ-39 (%)**	12.61	13.50	42	6.43	5.78	88	p = 0.017	*

SD, standard deviation; M, male; F, female; n, sample size; MDS-UPDRS, Movement Disorder Society - Unified Parkinson's Disease Rating Scale; RBDSQ, REM Sleep Behavior Disorder (RBD) Screening Questionnaire; BSIT, Brief Smell Identification Test; BDI-I, Beck Depression Inventory; SAS, Starkstein Apathy Scale; PDQ-39, Parkinson's disease questionnaire 39-item. *Significant at the unadjusted 5% level ($p \leq 0.05$) (two-tailed); **Significant at the Bonferroni-adjusted 5% level ($p \leq 0.05/7$) (two-tailed).

After matching for age and sex, we compared 88 P-PD participants with 88 control subjects (Supplementary Figure 1C). Confirming successful matching, the groups did not differ significantly in sex (p = 1.000) or age (p = 0.648). They did not differ significantly in years of education (p = 0.058). After multiple testing correction, the P-PD group presented significantly higher SAS (p < 0.001), BDI-I (p = 0.001), MDS-UPDRS I & II (p = 0.002, p < 0.001, respectively), and nominally significant lower score for PDQ-39 (p = 0.017) compared to the matched control subjects.

Supplementary Table 2. Results of neuropsychological assessments for prodromal PD (P-PD) compared to the control group.

**Variable**	**Descriptive statistics**						**Significance**	
	**Prodromal PD** n = 88			**Control** n = 88			**Prodromal PD vs. Control**	
	Mean	*SD*	*N*	*Mean*	*SD*	*N*		
CUPRO								
Intermediate Score 1 (IS1) (/3)	2.22	1.07	88	2.40	0.94	88	p = 0.273	
Intermediate Score 2 (IS2) (/3)	2.06	1.14	88	2.63	0.78	88	p < 0.001	**
CUPRO Total score (/6)	4.27	1.94	88	5.02	1.49	88	p = 0.010	*
Montreal Cognitive Assessment (MoCA) (/30)	25.22	3.41	88	27.09	2.55	88	p < 0.001	**
Trail-Making-Test								
Part A (TMT-A) (s)	43.98	18.73	48	38.42	16.37	88	p = 0.071	
Part B (TMT-B) (s)	116.4	57.99	48	82.83	28.05	88	p < 0.001	**
Delta-TMT (TMT-B) – (TMT-A)	72.46	51.24	48	44.41	29.43	88	p < 0.001	**

SD, standard deviation; CERAD, Consortium to Establish Registry for Alzheimer Disease. *Significant at the unadjusted 5% level (p ≤ 0.05) (two-tailed); **Significant at the Bonferroni-adjusted 5% level (p ≤ 0.05/7) (two-tailed).

Significant group differences were found in cognition. The P-PD group presented significantly lower scores in CUPRO Intermediate Score 2 (IS2) (p < 0.001), MoCA (p < 0.001), TMT-B and Delta-TMT scores (p < 0.001) compared to the control group.

Conclusion

In the present study, we found significant differences for the Cube copying task (initial scoring on 1 point [2]) but not for the Interlocking Pentagon copying task. To be able to interpret if this observed difference in the Cube copying task is due to an impaired visuo-constructive functioning or due to retrograde procedural memory deficit, we performed additional analyses in Set 2 (Supplementary Figure 1C). We applied the CUPRO evaluation system allowing the separate assessment of the Cube drawing procedure (CUPRO-IS1), suggestive of retrograde procedural memory, and of the final result of the Cube (CUPRO-IS2), suggestive of visuo-constructive

functions. No significant differences were observed for retrograde procedural memory (CUPRO-IS1), while visuo-constructive functions were affected in the P-PD group (CUPRO-IS2). This is consistent with previous findings, stating that the Cube copying assessment is more sensitive than the Interlocking Pentagon assessment, most likely related to the Cubes' greater complexity [3]. No significant difference had been observed for visuo-spatial judgment. Until now, visuo-cognitive abilities have only been investigated sparsely [4,5] and findings are still controversial [6–9].

REFERENCES

[1] Pauly L, Pauly C, Hansen M, Schröder VE, Rauschenberger A, Leist AK, Krüger R (2022) Retrograde procedural memory in Parkinson's disease: a cross-sectional, case-control study. *J Parkinsons Dis* **12**, 1013–1022.

[2] Nasreddine ZS, Phillips NA, Bedirian V, Charbonneau S, Whitehead V, Collin I, Cummings JL, Chertkow H (2005) The Montreal Cognitive Assessment, MoCA: a brief screening. *J Am Geriatr Soc* **53**, 695–699.

[3] Alty JE, Cosgrove J, Jamieson S, Smith SL, Possin KL (2015) Which figure copy test is more sensitive for cognitive impairment in Parkinson's disease: Wire cube or interlocking pentagons? *Clin Neurol Neurosurg* **139**, 244.

[4] Liepelt-Scarfone I, Ophey A, Kalbe E (2022) Cognition in prodromal Parkinson's disease. *Prog Brain Res* **269**, 93-111.

[5] Fengler S, Liepelt-Scarfone I, Brockmann K, Schäffer E, Berg D, Kalbe E (2017) Cognitive changes in prodromal Parkinson's disease: A review. *Mov Disord* **32**, 1655–1666.

[6] Pausch C, Schomburg R, Wagenpfeil S, Wollenweber FA, Bayer C, Fassbender K, Behnke S (2016) Neuropsychological impairment in prodromal Parkinson's disease. *J Neurol Sci* **371**, 117–120.

[7] Fantini ML, Farini E, Ortelli P, Zucconi M, Manconi M, Cappa S, Fernini-Strambi L (2011) Longitudinal study of cognitive function in idiopathic REM sleep behavior disorder. *Sleep* **34**, 619–625.

[8] Weintraub D, Chahine LM, Hawkins KA, Siderowf A, Eberly S, Oakes D, Seibyl J, Stern MB, Marek K, Jennings D (2017) Cognition and the course of prodromal Parkinson's disease. *Mov Disord* **32**, 1640–1645.

[9] Chastan N, Bair W-N, Resnick SM, Studenski SA, Decker LM (2019) Prediagnostic markers of idiopathic Parkinson's disease: Gait, visuospatial ability and executive function. *Gait Posture* **68**, 500–505.
